# Alternative futures of dissolved inorganic nitrogen export from the Mississippi River Basin: influence of crop management, atmospheric deposition, and population growth

**DOI:** 10.1007/s10533-017-0331-z

**Published:** 2017-04-10

**Authors:** Michelle L. McCrackin, Ellen J. Cooter, Robin L. Dennis, John A. Harrison, Jana E. Compton

**Affiliations:** 1 School of the Environment, Washington State University, Vancouver, WA USA; 2 Office of Research and Development, National Exposure Research Lab, U. S. Environmental Protection Agency, Research Triangle Park, NC USA; 3 Office of Research and Development, National Health and Environmental Effects Research Laboratory, Western Ecology Division, U. S. Environmental Protection Agency, Corvallis, OR USA; 4 Present Address: Baltic Sea Centre, Stockholm University, Stockholm, Sweden

**Keywords:** Gulf of Mexico, Mississippi River Basin, Dissolved inorganic nitrogen, Nitrogen export model, Tile drainage

## Abstract

Nitrogen (N) export from the Mississippi River Basin contributes to seasonal hypoxia in the Gulf of Mexico (GOM). We explored monthly dissolved inorganic N (DIN) export to the GOM for a historical year (2002) and two future scenarios (year 2022) by linking macroeonomic energy, agriculture market, air quality, and agriculture land management models to a DIN export model. Future scenarios considered policies aimed at encouraging bioenergy crop production and reducing atmospheric N-emissions, as well as the effect of population growth and the states’ infrastructure plans on sewage fluxes. Model-derived DIN export decreased by about 9% (from 279 to 254 kg N km^−2^ year^−1^) between 2002 and 2022 due to a 28% increase in area planted with corn, 24% improvement in crop N-recovery efficiency (NRE, to 0.52), 22% reduction in atmospheric N deposition, and 23% increase in sewage inputs. Changes in atmospheric and sewage inputs had a relatively small effect on DIN export and the effect of bioenergy crop production depended on nutrient management practices. Without improved NRE, increased production of corn would have increased DIN export by about 14% (to 289 kg N km^−2^ year^−1^) between 2002 and 2022. Model results suggest that meeting future crop demand while reducing the areal extent of hypoxia could require aggressive actions, such improving basin-level crop NRE to 0.62 or upgrading N-removal capabilities in waste water treatment plants beyond current plans. Tile-drained cropland could contribute up to half of DIN export; thus, practices that reduce N losses from tile drains could also have substantial benefit.

**Electronic supplementary material:**

The online version of this article (doi:10.1007/s10533-017-0331-z) contains supplementary material, which is available to authorized users.

## Introduction

Nitrogen enrichment contributes to eutrophication of surface waters, which is one of the greatest stressors for freshwater and coastal marine ecosystems globally (Howarth et al. 2011). Eutrophication is associated with the increased duration, frequency, and extent of harmful algal booms and hypoxic “dead zones” (areas that lack sufficient oxygen to support life). The largest hypoxic zone in the United States (US) and the second-largest worldwide is in the northern Gulf of Mexico (GOM), adjacent to the Mississippi River. This hypoxic zone varies annually in size from 40 km^2^ to >20,000 km^2^ due to climate and riverine N inputs (Turner et al. 2006). Ecological consequences include altered spatial distribution and reduced reproductive success of fish and benthic organisms (USEPA 2013).

The Gulf of Mexico Hypoxia Task Force (HTF) was established in 1997 to understand the causes and effects of eutrophication and coordinate activities to reduce the size, severity, and duration of hypoxia (USEPA 2013). Since then, the HTF has developed action plans to reduce riverine nutrient export to the GOM from agriculture, atmospheric deposition, and sewage effluent. However, despite efforts to reduce N delivery to the GOM, current total N (TN) export in the MRB is about double the target (USGS 2015). A recent assessment extended the timeframe to meet the areal hypoxia goal (<5000 km^2^) to 2035 and established an interim target of a 20% reduction in riverine N export by 2025 (compared to the 1980–1996 baseline period) (USEPA 2014b). This situation is not unique; indeed, mitigating the effects of eutrophication through nutrient reductions is proving difficult in many regions, such as Chesapeake Bay, Lake Erie, and the Baltic Sea (HELCOM 2016; USEPA 2016a, b).

Achieving nutrient reductions can be complicated by the unintended consequences of policies in other sectors. For example, programs that encourage the production of bioenergy crops have the potential to increase fertilizer inputs to cropland (Lambert et al. 2016). On the other hand, improvements in air quality standards aimed at protecting human health have substantially reduced deposition of N oxides and could have ameliorating effects on N transport in the MRB (Lloret and Valiela 2016). Lastly, population growth and urbanization could increase the number of people served by centralized wastewater treatment plants (WWTPs) with the potential to increase nutrient inputs to surface waters and reverse many of the gains of the Clean Water Act (Adler 2013).

While hypoxia management goals for the MRB address TN, nitrate (plus nitrite; hereafter as nitrate or NO_3_^−^) is particularly important because previous work has found that NO_3_^−^ export in the month of May is the best predictor of the areal extent of summer hypoxia (Turner et al. 2006); NO_3_^−^ constitutes about two-thirds of TN export and 98% of dissolved inorganic N (DIN) export (Aulenbach et al. 2007). In this study, we estimated monthly export of dissolved inorganic N (DIN) to the GOM before (year 2002) and after (year 2022) the implementation of policies related to bioenergy crop production and air quality. We also considered the effect of population growth on sewage inputs during this 20-year period. To simulate the policy effects, we used a novel approach of linking macroeconomic energy and agriculture market models with air quality and agriculture land management models. We then used output from those models as inputs for NEWS2_MRB_-DIN, a nutrient export model.

NEWS2_MRB_-DIN is a new model that builds on the Global Nutrient Export from WaterSheds (NEWS2) model (Mayorga et al. 2010) in two major respects. First, we estimated monthly DIN export, in contrast to previous annual-scale applications. Second, we modified model structure to account for N transport through tile drains, a factor not explicitly included in past NEWS2 models. Tile-drain systems have been identified as an important source of NO_3_^−^ in the MRB (Randall and Mulla 2001), however, the magnitude of the contribution cannot be empirically measured at large river-basin-scales. In the absence of such measurements, models capable of source apportionment, such as NEWS2_MRB_-DIN can provide first-order estimates.

## Methods

**Fig. 1:**
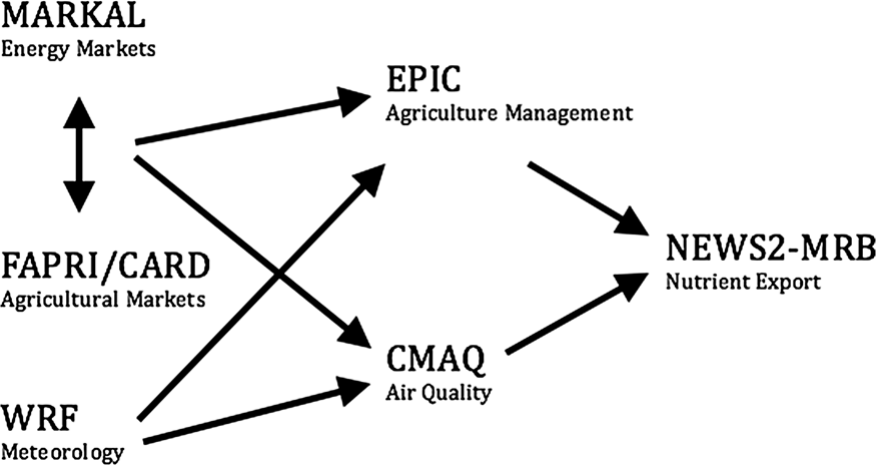
Diagram of loose coupling of models used in this study

Model inputs to NEWS2_MRB_-DIN are from the “upstream” linking of the MARKAL (macroeconomic energy) and FAPRI/CARD models (agricultural market) to the CMAQ (air quality) and EPIC models (agriculture land management, Fig. 1) (ETSAP 2011; Fabiosa et al. 2010). There are no feedbacks between downstream and upstream models. Here we describe NEWS2_MRB_-DIN in detail; summaries of CMAQ and EPIC are included in Supplemental Materials and are described in detail elsewhere (Cooter et al. 2012; Williams et al. 2012).

**Fig. 2:**
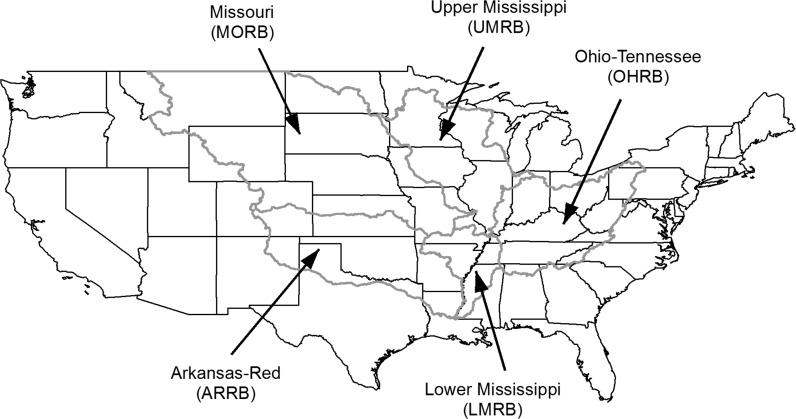
Delineation of the Mississippi River Basin and major sub-basins (*blue lines*). Basin abbreviations are in *parentheses*. (Color figure online)

We applied NEWS2_MRB_-DIN) to the entire MRB and its major sub-basins: Arkansas-Red River basin (ARRB), Ohio-Tennessee River basin (OHRB), Lower Mississippi River basin (LRMB), Missouri River basin (MORB), and Upper Mississippi River basin (URMB) (Fig. 2).

### NEWS2_MRB_-DIN model overview

NEWS2_MRB_-DIN is a deterministic, river-basin-scale model based on NEWS2-DIN (Mayorga et al. 2010), which estimates DIN export at river mouths. Total N inputs to land and DIN exports from each basin occur in the same month and changes in N storage within basins are not taken into account. Sources and sinks of N are distributed homogeneously in each basin. NEWS2_MRB_-DIN considers different point and diffuse sources and several retention pathways on land and in rivers. Monthly DIN export (kg N km^−2^ mo^−1^) for the MRB and its major sub-basins was estimated as:

1 $${\text{DIN}} = \left( {\left( {\left( {{\text{TN}}_{\text{diff}} - {\text{ TN}}_{\text{tile}} } \right) \times {\text{FE}}_{\text{ws}} } \right) + \left( {\left( {{\text{TN}}_{\text{sew}} \times {\text{FE}}_{\text{sew}} } \right) + {\text{TN}}_{\text{tile}} } \right)} \right) \times {\text{FE}}_{\text{riv}}$$

TN_diff_ represents diffuse sources of TN that are available for transport to the river network:

2 $${\text{TN}}_{\text{diff}} = {\text{TN}}_{\text{dep}} + \left( {\left( {{\text{TN}}_{\text{BNFcrop}} + {\text{TN}}_{\text{fert}} } \right) \times {\text{NRE}}} \right) + {\text{TN}}_{\text{BNFnat}} + {\text{TN}}_{\text{sep}}$$

where TN_dep_ is atmospheric deposition, TN_BNFcrop_ is biological N-fixation (BNF) by crops, TN_fert_ is organic and inorganic fertilizer application, TN_BNFnat_ is BNF in natural and non-agricultural areas, and TN_sep_ is leakage from septic systems (all in kg N km^−2^ mo^−1^). Nitrogen recovery efficiency (NRE) is the fraction of fertilizer and crop BNF removed from fields by crop harvest.

TN_tile_ is excess fertilizer not taken up by crops on tile-drained fields:

3 $${\text{TN}}_{\text{tile}} = {\text{TN}}_{\text{fert}} \times \left( { 1 { }{-}{\text{ NRE}}} \right) \times {\text{Tile}}_{\text{area}}$$

where Tile_area_ is the fraction of basin area where tile drains are present.

The fraction of diffuse N sources transported from soils to the river network (FE_ws_) was modeled as a function of runoff and temperature as:

4 $${\text{FE}}_{\text{ws}} = {\text{FE}}_{\text{RO}} \times \left( { 1 { } - {\text{ F}}_{\text{temp}} } \right)$$

where FE_RO_ is the fraction of TN exported from land to the river mouth as DIN as a function of runoff and F_temp_ is the fraction of TN retained in the basin as a function of temperature. FE_RO_ was calculated as:

5 $${\text{FE}}_{\text{RO}} = {\text{b}} \times \left( {{\text{R}}^{\text{a}} } \right)$$

where R is runoff (m mo^−1^) and a and b are calibrated parameters that define the shape of the relationship between R and FE_RO_. F_temp_ was:

6 $${\text{F}}_{\text{temp}} = {\text{d}} \times \left( {{\text{T}}/ 100} \right)^{\text{c}}$$

where T is average seasonal air temperature (°C + 8, to eliminate negative values) and c and d are calibrated parameters that define the shape of the relationship between T and F_temp_. Air temperature is divided by 100 so that values are similar in magnitude to runoff (McCrackin et al. 2014). The exponential relationship between temperature and F_temp_ reflects temperature-dependent processes such as respiration (Green et al. 2004).

TN_sew_ is N in human excrement that enters centralized wastewater treatment plants (WWTP). FE_pnt_ is the fraction of TN_sew_ that is DIN (see also Supplemental Material).

The fraction of diffuse and point N sources transported by rivers to the coastal zone, FE_riv_, was estimated as:

7 $${\text{FE}}_{\text{riv}} = \left( { 1- {\text{F}}_{\text{deN}} } \right) \times \left( { 1- {\text{F}}_{\text{Qrem}} } \right) \times \left( { 1- {\text{F}}_{\text{res}} } \right)$$

where riverine sinks are denitrification in the river channel (F_deN_), consumptive water use (F_Qrem_), and denitrification and sedimentation in reservoirs (F_res_; see also Supplemental Material).

We present contributions of different N sources to DIN export (tile-drained agriculture, non-tile drained agriculture, atmospheric deposition, sewage, and background BNF) on an annual basis because transient storage across months could obscure monthly source contributions.

### Description of scenarios

We examine a historical year (2002, hereafter as 2002_HIST_) and two future scenarios (2022_BASE_ and 2022_CROP_). Year 2002_HIST_ reflects environmental conditions prior to the implementation of air quality standards that have reduced emissions of N oxides (*ca*. 2004). It also pre-dates the implementation of programs that encouraged the production of bioenergy crops, some of which competed with demand for food and livestock feed production (*ca*. 2007).

The year 2022 scenarios are not predictions of future events, but intended to provide insight on potential consequences resulting from changes in land-based N management and a larger population. NEWS2_MRB_-DIN inputs for year 2022 are outputs from the EPIC and CMAQ models. The 2022_BASE_ scenario includes technology- and intensification-driven increases in crop yields and increased ambient CO_2_ concentrations (Table 1). In 2022_BASE_, crop production is met through these trends without any change from 2002_HIST_ cropping area or crop distribution (Table 1). Atmospheric N emissions used in 2022_BASE_ reflect reductions resulting from the full implementation of the Clean Air Interstate Rule and Tier 2 light-duty vehicle standards (USEPA 2010).

**Table 1:** Summary of key model drivers for year 2002 and two 2022 scenarios for the total Mississippi River Basis (MRB)

Model drivers	2002_HIST_	2022_BASE_	2022_CROP_	Sources
Agriculture				
Land management	*ca*. 2002, all conservation tillage	Intensification, conservation tillage	2022_BASE_ plus extensification	Elobeid et al. (2013); EPIC simulation
Corn ethanol production, billion gallon ethanol	Corn Grain: 0 Corn Stover: 0	Corn Grain: 12.3 Corn Stover: 0	Corn Grain: 18.4 Corn Stover: 10.7	2022_BASE_: https://www.gpo.gov/fdsys/pkg/FR-2010-03-26/pdf/2010-3851.pdf, Table IV.A.2-1 2022_CROP_: Elobeid et al. (2013)
Organic and inorganic fertilizer (TN_fert_) (kg N km^−2^ year^−1^)	3285	3578	3513	EPIC simulation
Crop BNF (TN_BNFcrop_), (kg N km^−2^ year^−1^)	1715	1753	1744	EPIC simulation
Atmospheric CO_2_, ppm	372	412	412	http://www.esrl.noaa.gov/gmd/ccgg/trends
Basin area where tile drains are present (%)	8	8	8	Sugg (2007)
Atmospheric N deposition				
Atmospheric N deposition (TN_dep_) (kg N km^−2^ year^−1^)	905	707	707	CMAQ simulation
Human sewage				
TN in human excrement (TN_sew_), kg N person^−1^ year^−1^	6.1	6.1	6.1	Sobota et al. (2013)
Population (million people)	69.7	84.2	84.2	US Census (2014)
Population connected to municipal sewage systems (%)	69	70	70	USEPA (2008)
TN exported from WWTP as DIN (FE_sew_) (%)	45	41	41	USEPA (2008), Van Drecht et al. (2009)

Values for sub-basins may differ from those used for the MRB (see also Supplemental Fig. S1)

Crop yields in 2022_CROP_ reflect the same technological improvements, intensification patterns, and CO_2_ concentrations as 2022_BASE_ and also crop extensification (e.g. shifting crop locations and management) to meet increased demand for starch and cellulosic biofuel feedstock (Tables 1, 2). Atmospheric N deposition rates used in 2022_CROP_ build upon those in 2022_BASE_ and include additional emissions from increased starch and cellulosic biofuel production (see also Supplemental Material). For both year 2022 scenarios, rates of sewage production reflect the projected 20% increase in population over 2002 (US Census 2014) and the states’ reported plans for sewage infrastructure (USEPA 2008).

**Table 2:** Nitrogen removal efficiency (NRE) and area planted for the seven largest crops (in terms of area planted) for 2002 and two 2022 scenarios for the Mississippi River Basin

Crop	2002_HIST_		2022_BASE_		2022_CROP_	
	NRE	Area planted (1000 ha)	NRE	Area planted (1000 ha)	NRE	Area planted (1000 ha)
Corn	0.51	22,275	0.65	22,275	0.62	28,432
Soybeans	0.30	21,785	0.35	21,785	0.35	21,591
Wheat	0.40	10,161	0.48	10,161	0.48	9899
Sorghum	0.44	1687	0.52	1687	0.52	731
Cotton	0.53	1793	0.71	1793	0.69	546
Barley	0.39	486	0.48	486	0.48	338
Oats	0.41	384	0.44	384	0.44	130
Overall	0.42	117,921	0.52	117,921	0.52	117,921

Columns do not sum because not all crops are presented

In addition to the described scenarios, we conducted an ad hoc analysis to estimate the effect of upgrading all WWTP to tertiary capabilities on DIN export. We also estimated the extent of improvement in NRE needed to achieve the interim target of a 20% reduction in DIN export (the HTF interim target). These analyses were performed by changing inputs to NEWS2_MRB_-DIN and did not capture potential feedbacks from the upstream models.

### Calibration and statistical analysis

For development and calibration of NEWS2_MRB_-DIN, we averaged monthly measurement-based DIN export (in kg N km^−2^ mo^−1^, sum of ammonium and nitrate) and runoff data (in m mo^−1^) for the period 1993 to 2002 (USGS 2013). Sub-basin N export was estimated by summing export for downstream stations and subtracting export for upstream stations, which could produce negative values arising from measurement error at monitoring stations or due to a net N sink within a sub-basin (Aulenbach et al. 2007). Indeed, average DIN export for the LMRB was negative for the period 1993 to 2002 and, thus, was excluded from model runs because NEWS2_MRB_-DIN cannot accommodate negative values. We estimated DIN export from the LMRB as the difference between modeled DIN export for the MRB and the sum of modeled DIN export for the other sub-basins.

The values of unknown parameters (a, b, c, and d) in Eqs. (5) and (6) allowed for the best fit of modeled DIN export to measurement-based DIN export. The calibration routine was repeated 1000 times and the “best estimate” parameters were the median of the resampling iterations (Table S1). All months for the MRB and its sub-basins for 2002_HIST_ were calibrated simultaneously using a resampling approach where 80% of monthly budgets for all basins were randomly selected and the best-fit parameters were estimated by maximizing Nash–Sutcliffe Efficiency (NSE):

8 $${\text{NSE}} = 1 { }{-}\frac{{\sum\nolimits_{{{\text{i}} = 1}}^{\text{n}} {(Obs_{i} - Mod_{i} )^{2} } }}{{\sum\nolimits_{{{\text{i}} = 1}}^{\text{n}} {(Obs_{i} - \overline{Obs}_{i} )^{{^{2} }} } }}$$

where Mod_i_ and Obs_i_ are the model-predicted and average measurement-based estimates, respectively, for the _i_th basin. NSE values between 0 and 1 indicate model predictions are better than simply using the mean of measurements to predict DIN export and values >0.5 are considered to be satisfactory (Moriasi et al. 2007).

We assessed the percent bias (PBIAS) of NEW2_MRB_-DIN estimates for 2002_HIST_ using:

9 $${\text{PBIAS}} = \frac{{\sum\nolimits_{{{\text{i}} = 1}}^{\text{n}} {(Obs_{i} - Mod_{i} ) \times 100} }}{{\sum\nolimits_{{{\text{i}} = 1}}^{\text{n}} {(Obs_{i} )} }}$$

Values for PBIAS less than ±70% are considered satisfactory (Moriasi et al. 2007).

During model development, we preserved the original NEWS2-DIN structure that did not account for tile drains. Preliminary parameter fitting found that model performance was poor (NSE = 0.4), however, including tile drains (as in Eqs. 1 and 3) resulted in substantially improved model fit. Initial model runs with tile drains included a parameter to partition between surface and tile-drain (sub-surface) N loss from cropland. The partitioning parameter did not improve model fit and was not included in the final model, which treats all N losses in tile-drained areas as subsurface losses that bypass retention mechanisms (e.g. represented in FE_ws_).

### NEWS2_MRB_-DIN Model Inputs

We constructed monthly N budgets for the MRB and its sub-basins to account for inputs of organic and inorganic fertilizers, crop BNF, crop NRE, atmospheric deposition, background BNF, and human sewage (Fig. S1) (Mayorga et al. 2010). Other data inputs to NEWS2_MRB_-DIN included areal cover of tile drains, runoff, and temperature.

Inorganic and organic fertilizer applications (TN_fert_) and crop BNF (TN_BNFcrop_) for 2002_HIST_, 2022_BASE_, and 2022_CROP_ were obtained from EPIC simulations. We used 3-month running averages for TN_fert_ applications for model input after preliminary model runs revealed substantial improvement in model performance over using monthly inputs. Three-month running average values likely reflect transient storage or differences between EPIC regional estimates and site-specific fertilization practices.

We defined N recovery efficiency (NRE) as the fraction of fertilizers and crop BNF removed from the field at harvest, which we assumed to be roughly equivalent to the annual EPIC Harvest Index (Table 2). An increase in NRE between years implies that a greater portion of N is removed in harvest and a greater yield per harvested area, which can occur with improved harvesting technology or increased harvestable product per plant. Increased NRE can also be achieved through increased N-use efficiency (NUE) resulting from “right time, right place, and right amount” best management practices. The NRE values used here represent a combination of production technology and on-field management factors. Likewise, crop production changes between 2002 and 2022 are projected by the integrated market model (Fig. 1) and, from an agricultural economics perspective, reflect future trends in technology, intensification (both year 2022 scenarios) and extensification (2022_CROP_ only). Crop-specific NRE (Table 2) was weighted based on planting area to estimate basin-scale NRE, which was 0.42, 0.52, and 0.52 in the MRB for 2002_HIST_, 2022_BASE_, and 2022_CROP_, respectively. Monthly NRE was constant across 2002_HIST_, 2022_BASE_, and 2022_CROP_, recognizing this to be a simplification because NRE likely varies across the growing season for different crops (Bender et al. 2013). EPIC is available for download as part of the Fertilizer Emission Scenario Tool for CMAQ (FEST-Cv1.1) at http://www.cmascenter.org. Monthly rates of atmospheric N deposition (TN_dep_) for 2002_HIST_, 2022_BASE_, and 2022_CROP_ were from CMAQ version 5.0.2 with Bi-Directional Ammonia, which simulates the emission and subsequent deposition of ammonia resulting from fertilizer application. Other deposition sources include fossil-fuel and bioenergy crop combustion.

Background N inputs (TN_BNFnat_) were based on annual rates of BNF in natural and non-agricultural areas from the US Environmental Protection Agency EnviroAtlas (USEPA 2014a). These BNF estimates were for year 2006, the year closest to 2002 for which spatially explicit data were available; we assume these data appropriately represent year 2002 conditions. Annual BNF was disaggregated into monthly values in proportion to evapotranspiration for each basin (Ahn and Tateishi 1994). Background BNF rates were held constant between 2002_HIST_ and both 2022 scenarios to focus on effects on DIN export based on changes in anthropogenic N-sources.

Total N from human sewage (TN_sew_) was TN in human excrement multiplied by portion of the population connected (F_sewer_, Table 1, Supplemental Materials) to WWTP. The portion of TN in sewage from WWTP emitted to rivers as DIN (FE_sew_) was estimated based on the population served by primary, secondary, and tertiary treatment capabilities (USEPA 2008). Sewage N not removed by WWTP was discharged to river networks in effluent as a point source. The portion of the population not connected to WWTP was assumed to be connected to septic systems, which retained 54% of N in human excrement (Sobota et al. 2013) and exported the remainder as a diffuse source (TN_sep_). Sewage-related model inputs were the same for both 2022 scenarios.

The model structure for N transport by tile drains (TN_tile_) suggests that sub-surface drainage transports all N fertilizer applied in excess of crop uptake. We recognize this structure is a simplification because numerous modeling and field studies suggest that NO_3_^−^ transport through surface and subsurface pathways on tile-drained fields depends on factors such as tile drain spacing and depth, soil moisture, and slope (Williams et al. 2015). Here we include DIN export through tile drains as a first-order estimate for large river-basin scales. Tile_area_ (Eq. 3) was the same in 2002 and both 2022 scenarios and was obtained from Sugg (2007), who used soil and land-cover maps to estimate the percent of land area with tile drains (Fig. S7). TN_tile_ was estimated on an annual basis for all basins and disaggregated into months in proportion to measurement-derived runoff to represent the flushing effect of runoff on transport though tile drains (Randall and Mulla 2001).

Riverine N export can vary dramatically from year-to-year because of weather and hydrology (Davis et al. 2014). Given our short 20-year study horizon, we held runoff and temperatures constant between years 2002 and 2022 to focus on the effects of shifts in crop production, reductions in atmospheric deposition, and a larger population on riverine input to the GOM. NEWS2_MRB_-DIN, used monthly runoff estimated from discharge measurements for the period 1993 to 2002 (USGS 2013). Average monthly air temperatures were from PRISM Climate Group (2015).

## Results

### Model performance

**Fig. 3:**
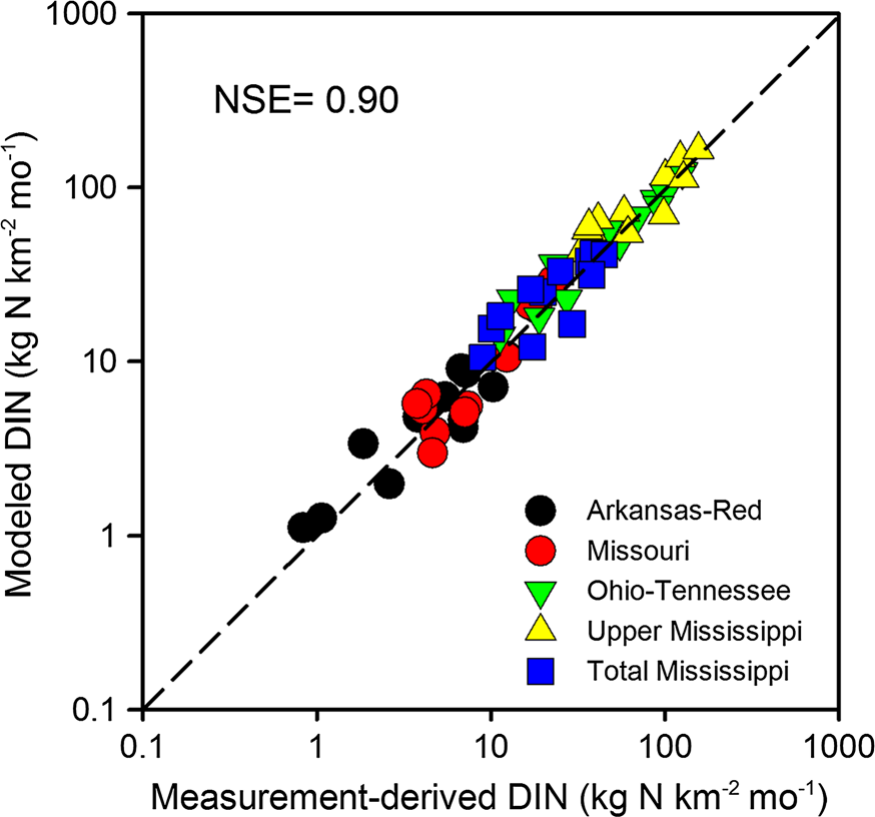
Comparison of measured and model-derived dissolved inorganic nitrogen (DIN) yields (kg N km^−2^ mo^−1^) for the Mississippi River Basin and four major sub-basins. NSE is Nash–Sutcliffe efficiency. The *diagonal line* indicates 1:1

**Fig. 4:**
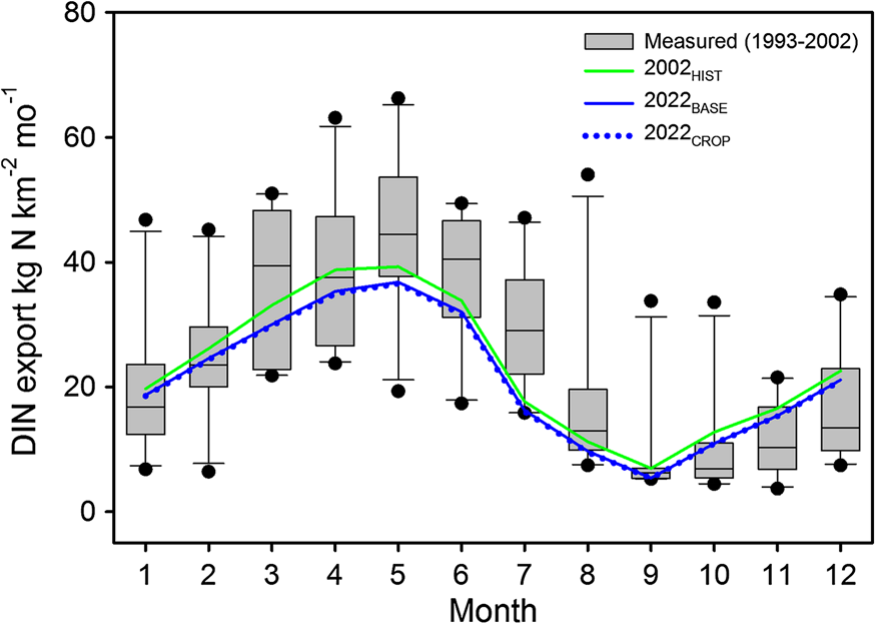
*Upper panel* comparison of measured (*gray bars*) and model-derived (*lines*) dissolved inorganic nitrogen (DIN) export (kg N km^−2^ mo^−1^) for the Mississippi River Basin. Box plot shows median value (*horizontal lines*), 25th and 75th percentile (*box outline*), 10th and 90th percentile (*error bars*), and 5th and 95th percentile (*solid points*)

Nash–Sutcliffe Efficiency for the MRB and sub-basins was 0.90 (Fig. 3). The scatter of observed and modeled DIN export around the 1:1 line indicated no obvious bias. Model-derived DIN export for 2002_HIST_ was within the range of measurement-derived export on an annual basis, with percent bias (PBIAS) ranging from 6% for the MRB to -50% for the AARB (Table 3; Figs. 4, S2, S3, S4, S5). On a monthly basis, however, NEWS2_MRB_-DIN overestimated DIN export for the AARB for April to June and October to December, with PBIAS ranging from 75% in May to 154% in November. For the MRB and its other sub-basins, PBIAS was <±70% for all months.

**Table 3:** Percent bias (PBIAS) of NEWS2_MRB_-DIN model output for 2002_HIST_ for the MRB and its sub-basins

	Mississippi River Basin	Arkansas-Red River Basin	Missouri River Basin	Ohio-Tennessee River Basin	Upper Mississippi River Basin
January	−1	−4	−44	−10	2
February	4	7	−53	−9	−6
March	−9	26	−51	−14	−21
April	0	98	−34	−18	−14
May	−12	75	−22	−32	−23
June	−11	82	−17	−19	−22
July	−40	0	−47	−19	−34
August	−35	49	−59	−11	−27
September	−23	37	−67	−8	−10
October	27	154	−17	42	14
November	47	83	0	24	20
December	33	64	2	−1	25
Annual	6	−50	34	13	15

Positive and negative values indicate that model-derived export is greater than and less than, respectively, average measurement-based export for 2002

### Future dissolved inorganic nitrogen export

Land-based N inputs used in NEWS2_MRB_-DIN decreased by about 11% between 2002_HIST_ and 2022_CROP_ because agriculture N inputs increased 7%, NRE by crops improved 24%, atmospheric N deposition decreased by 22%, and sewage inputs increased by 50%. Together, this overall decline in land-based N inputs decreased annual DIN export by 8% between 2002_HIST_ and 2022_BASE_, from 279 to 256 kg N km^−2^ year^−1^, respectively. Between 2022_BASE_ and 2022_CROP_, the area planted with corn increased by 28% while agricultural N inputs decreased by about 1% because extensification onto poorer quality soils lowered crop N demand. In addition, soybean production, which requires little or no N inputs, was maintained to meet economic demand and replaced other crops that require higher N inputs (Tables 1, 2). Annual DIN export decreased 2 kg N km^−2^ year^−1^ between 2022_BASE_ and 2022_CROP_ to 254 kg N  km^−2^ year^−1^ because of these reduced agricultural N inputs.

We isolated the effects of policies aimed at encouraging bioenergy crop production from those designed to improve air quality as well as population-related changes in sewage on DIN yield between 2002 and 2022. Just considering changes in assumptions relating to agriculture, DIN decreased 22 kg N km^−2^ year^−1^ between 2002 and 2022_CROP_. This change was composed of an increase of 13 kg N km^−2^ year^−1^ resulting from shifts in planted crops and extensification that was more than counterbalanced by decreases of 35 kg N km^−2^ year^−1^ due to a 24% improvement in NRE (from 0.42 to 0.52). In other words, DIN yield would have increased 13 kg N km^−2^ year^−1^ between 2002 and 2022_CROP_ due to bioenergy crop production had there not been concurrent improvements in NRE. By comparison, the effect of air quality policies was smaller and resulted in a reduction of 6 kg N km^−2^ year^−1^ between 2002 and both year 2022 scenarios. Lastly, sewage sources increased DIN by 3 kg N km^−2^ year^−1^ over the study period. This change was composed of composed of an increase of 6 kg N km^−2^ year^−1^ due to a 20% larger population that was counterbalanced by a decrease of 3 kg N km^−2^ year^−1^ based on the states’ plans for improving N-removal efficiency of WWTP from 55% in 2002 to 59% in 2022. Overall, improvements in crop NRE had the largest potential impacts on the N delivery to the coast, with substantial reductions possible from these improvements (Table 3).

We conducted additional analyses of the effects of upgraded WWTP capabilities and reductions in fertilizer inputs on DIN export by modifying NEWS2_MRB_-DIN model input. Improving sewage N removal-efficiency to 80% could reduce DIN export in the MRB by about 5% (about 13 kg N km^−2^ year^−1^ or 40 kt N year^−1^) compared to 2002_HIST_, despite a larger population. Achieving a 20% reduction in average annual DIN export between 2002 and 2022 (56 kg N km^−2^ year^−1^ or 177 kt N year^−1^) would require a 43% improvement in basin-scale NRE to 0.62 under 2022_CROP_ levels of N inputs.

On an annual basis, agricultural inputs (organic and inorganic fertilizer and crop BNF on tile-drained and non-tile drained cropland) were the greatest source of DIN for the MRB and sub-basins for 2002_HIST_ and both 2022 scenarios (Tables 4, 5). For the OHRB, UMRB, and MRB, N from tile-drained cropland represented 46, 68, and 46% of total DIN export, respectively, across the three modeled years. In contrast, N from tile-drained crop land was 1% and 18% of annual DIN export for the ARRB and MORB, respectively. Sewage was the second greatest source of N for all basins (8–21% of annual DIN, depending on basin and year), followed by atmospheric deposition and background BNF (5–19% and 1–13% of annual DIN, respectively).

**Table 4:** Percent contribution based on median calibrated parameters for different sources of dissolved inorganic nitrogen export for the Mississippi River Basin and major sub-basins

2002_HIST_	Mississippi River Basin (%)	Arkansas-Red River Basin (%)	Missouri River Basin (%)	Ohio-Tennessee River Basin (%)	Upper Mississippi River Basin (%)
Tile-drained agriculture	47	1	18	46	68
Non-tile drained agriculture	28	48	45%	27	18
Atmospheric deposition	10	19	13	12	5
Human sewage	10	21	16	10	8
Background	5	12	9	5	1
2022_BASE_					
Tile-drained agriculture	46	1	17	45	68
Non-tile drained agriculture	28	52	43	28	17
Atmospheric deposition	8	15	11	9	5
Human sewage	12	20	20	12	9
Background	6	13	9	6	1
2022_CROP_					
Tile-drained agriculture	45	1	16	45	67
Non-tile drained agriculture	28	52	43	28	17
Atmospheric deposition	8	15	11	9	5
Human sewage	12	20	20	13	9
Background	6	13	9	6	1

**Table 5:** Contribution based on median calibrated parameters for different sources of dissolved inorganic nitrogen export for the Mississippi River Basin and major sub-basins (kg N km^−2^ year^−1^)

2002_HIST_	Mississippi River Basin	Arkansas-Red River Basin	Missouri River Basin	Ohio-Tennessee River Basin	Upper Mississippi River Basin
Tile-drained agriculture	132	1	14	269	528
Non-tile drained agriculture	77	40	35	158	138
Atmospheric deposition	27	16	10	69	39
Human sewage	28	17	13	57	60
Background	14	10	7	31	10
2022_BASE_					
Tile-drained agriculture	119	1	14	249	480
Non-tile drained agriculture	74	41	35	154	123
Atmospheric deposition	21	12	9	47	34
Human sewage	32	16	16	69	63
Background	15	10	7	32	10
2022_CROP_					
Tile-drained agriculture	118	1	13	239	476
Non-tile drained agriculture	74	41	34	151	123
Atmospheric deposition	21	12	9	47	34
Human sewage	32	16	16	69	63
Background	15	10	7	32	10

## Discussion

Addressing nutrient losses from agriculture is a major component of plans to reduce the hypoxic area in the GOM (USEPA 2014b). Our analysis suggests there is potential for policies aimed at encouraging bioenergy crop production to increase DIN export in the MRB. Without improved NRE, increased corn production under 2022_CROP_ could increase DIN loads by 13 kg N km^−2^ year^−1^ (5%) compared to 2002_HIST_. These results are broadly similar with other modelling studies. For example, van Wijnen et al. (2015) estimated that increased production of bioenergy crops in Europe could increase DIN export by 9 to 25% to coastal areas of Europe, depending on the scenario. Donner and Kucharik (2008) estimated that a 20 to 30% increase in corn production in the MRB could increase annual DIN export 10 to 18%. Lastly, Secchi et al. (2011) estimated that a 15 to 28% increase in corn acreage could increase TN export in the UMRB by 4 to 9%.

Improvements in NRE could mitigate the effects of increased corn production and reduce DIN loads by 22 kg N km^−2^ y^−1^ (8%) between 2002_HIST_ and 2022_CROP_. The assumed improvement in NRE to 0.52 for both 2022 scenarios (Table 2) was not based on specific actions, but reflects general improvements that could be achieved by broad adoption of new crop hybrids or precision fertilizer application, for example. Reducing DIN export by 20% between 2002 and 2022 would require more substantial changes in farming practices – especially for corn – to increase basin-average NRE to 0.62. NRE for corn has a disproportionate contribution on overall NRE in the MRB; in 2022_CROP_, about one quarter of the area of planted crops is corn. Measured NRE for corn in the US averages 0.37, ranging from 0.31 to 0.49 depending on the cropping system (Cassman et al. 2002). The NRE assumptions for corn in 2022 (>0.6, Table 2) could be optimistic because this level of efficiency has not been observed across broad spatial scales (Ladha et al. 2005). However, dramatic efficiency gains are possible; for example, in the Netherlands fertilizer N inputs are the same as the 1960s but overall crop yields have doubled (Lassaletta et al. 2014).

The year 2022 scenarios suggest that planned improvements in WWTP infrastructure will not keep pace with population growth, resulting in an increase in sewage-related fluxes between 2002 and 2022 of about 3 kg N km^−2^ year^−1^ (1%). While agriculture is the dominant N source in the MRB (Tables 4, 5), there is also opportunity to reduce DIN export by addressing sewage effluent. A number of studies report success in reducing N loads to coastal areas by upgrading WWTP capabilities in areas such as Tampa Bay, Chesapeake Bay, and coastal waters of Denmark (Carstensen et al. 2006; Greening and Janicki 2006; Williams et al. 2010). We estimated that improving the N-removal efficiency of WWTPs in the MRB to 80% could reduce DIN by 14 kg N km^−2^ year^−1^ (5%) between 2002 and 2022 even with a 20% larger population.

Reductions in atmospheric N pollution can benefit human health and improve inland water quality (Davidson et al. 2012), however, these reductions have a relatively small impact on riverine DIN delivery to the GOM. Improved air quality standards reduce atmospheric N deposition in the MRB by about 200 kg km^−2^ year^−1^ between 2002 and 2022 (Table 1). The corresponding reduction of DIN export to the GOM was 6 kg km^−2^ year^−1^ (2% of 2002_HIST_) due to retention in the basin. Our approach only considers N deposition that is transferred by river networks to the GOM. Direct deposition of N to the GOM is estimated at <1% of the riverine N export from the MRB (Goolsby et al. 1999). There appears be greater opportunity to reduce DIN delivery to the GOM by addressing agriculture and sewage sources.

### Tile-drain systems

Nearly half of all artificially drained agriculture worldwide (including tile-drain systems) is found in the United States and European Union (Feick et al. 2005). While artificial drainage has enabled the cultivation of otherwise water-logged soils, it is a primary pathway for nutrient delivery to surface waters. Indeed, NEWS2_MRB_-DIN suggests that tile-drained agriculture contributes nearly half of DIN export in the MRB (Table 4). There were no appropriately scaled estimates to which we could compare these results. Large-scale models such as SPARROW and SWAT consider tile drainage but do not include it in source apportionment. The most recent SPARROW model for the MRB found that the density of tile drains increased total N export (Robertson and Saad 2013). SWAT models for the UMRB and OHTN found that sub-surface flows (including tile drains) accounted for 68% and 59%, respectively, of TN losses (USDA-NRCS 2012a, b). Studies of monitored fields and small basins suggest that tile drains contribute 54 to 95% of NO_3_^−^ export and 44 to 82% of TN export (Ikenberry et al. 2014; Tiemeyer et al. 2008; Williams et al. 2015). These model- and measurement-derived estimates suggest that tile drains can contribute a substantial portion of riverine N export, but are not strictly comparable to our results due to obvious differences in scales, assumptions, approaches, and N forms. Improved understanding of the importance of tile drains to N transport across large river basins is critical to inform policies aimed at reducing N losses from agriculture.

There could be greater opportunity to reduce N delivery to surface waters by modifying tile-drain systems than by implementing common conservation practices such grass buffer strips or strip tillage (Lemke et al. 2011). Potential modifications include the installation of bioreactors and constructed wetlands, which remove N through denitrification, and removable weir-boards that control in-field water levels to maximize root-water table interactions (Sunohara et al. 2015). Studies report that bioreactors and wetlands could remove 20 to 40% and 45 to 95%, respectively, of NO_3_^−^ from tile drains (Lemke et al. 2011; Moorman et al. 2015). Despite the strong potential for N removal, these practices are not widely used. A recent farmer survey in Illinois found that 23% of respondents were familiar with bioreactors and 43% of respondents were familiar with wetlands and controlled drainage, but <1% used bioreactors and only 6% used constructed wetlands or controlled drainage (David et al. 2015). Financial and operational constraints (e.g. taking land out of production to install wetlands or bioreactors) are substantial barriers. Funding for education and financial incentives in regions with dense tile-drain networks could encourage greater implementation of these practices.

### Uncertainties and future directions

Our approach could overstate the contribution of agriculture, including losses from tile-drained cropland, which together account for about 75% of DIN export for the MRB. If crop NRE is greatest early in the growing season (when fertilizer application rates are greatest), we might expect lower relative N losses to the environment on an annual basis compared to our approach, which assumes NRE is constant across months. To assess potential bias in NEWS2_MRB_-DIN, we examined annual source contribution estimates of other models. The SPAtially Referenced Regression On Watershed attributes (SPARROW) model suggested that agriculture contributes 74% (Smith et al. 1997), 70% (Alexander et al. 2008), or 60% (Robertson and Saad 2013) of TN export in the MRB. The Soil Water Assessment Tool (SWAT) model estimated that 80% of TN export in the MRB is from agriculture (White et al. 2014). Lastly, a recent application of NEWS2 (NEWS2_US_-TDN) to the contiguous US found that agriculture contributed 64% of total dissolved N export in the MRB (McCrackin et al. 2015). This contribution is lower than that found by NEWS2_MRB_-DIN, likely because agricultural N was 56% of the MRB N-budget for NEWS2_US_-TDN compared to 68% in this study (Fig. S1). Overall, the DIN export attributed to agriculture by NEWS2_MRB_-DIN falls within the range of other model estimates, but on the high side.

It was beyond the capabilities of NEWS2_MRB_-DIN to consider the effects of the scenarios on dissolved and particulate organic N sources forms, which constitute about one-third of TN flux in the MRB (Aulenbach et al. 2007). If DIN export decreases as suggested in 2022_CROP_, dissolved and particulate organic N forms will likely become a greater portion of TN flux. The ecological effect of such a shift in the GOM is not clear. Prior studies suggest, however, that DIN is more important in controlling the areal extent of the hypoxic zone than other N forms (dissolved organic or particulate) (Turner et al. 2006).

We held discharge and weather constant in order to isolate the effects of changes in land-based N management. Changes in the timing or magnitude of river discharge could have a greater effect on DIN export to the GOM than changes in land-based N management due to the accumulation of anthropogenic N in soils (Donner and Scavia 2007; Pellerin et al. 2014; Van Meter et al. 2016). In the last half of the twentieth century, MRB discharge increased 5.5% per decade. If this trend continues and monthly runoff increases by about 11% between 2002 and 2022, annual DIN export could increase by about 4% under 2022_CROP_ conditions. This estimate does not capture impacts of shifts in the timing of runoff between months, which are important to the development of hypoxia. In addition to increases in annual runoff, more extreme year-to-year variations, such as drought followed by flooding, could flush stored N from soils and result in anomalously high N concentrations and N export to the GOM (Davis et al. 2014). Donner and Scavia (2007) found that years with heavy precipitation could require a 55% reduction in riverine N concentrations to meet hypoxia targets. Future efforts could further link EPIC, CMAQ, and NEWS2_MRB_-DIN with downscaled climate and hydrologic models to explore the implications of variable runoff on DIN export.

## Conclusion

Despite two decades of effort, there has been an overall lack of progress in reducing the areal extent of the hypoxic zone in the GOM. The challenge of achieving nutrient load reductions is seen in many other regions worldwide and reflects the complexity of mitigating the effects of eutrophication by reducing land-based nutrient inputs. Meeting environmental goals can be helped or hindered by policies in other sectors and broader societal trends, such as population growth. Here we explored how policies aimed at increasing bioenergy crop production and improving air quality could affect future DIN export to the GOM by providing output from air quality and agriculture land management models as inputs to a DIN export model. We further considered how population growth and WWTP infrastructure plans could influence sewage effluent fluxes. The interaction of these drivers suggest alternative DIN outcomes, which complicate plans to reduce N export in the MRB.

Increased corn production has the potential to increase future DIN export, but could be mitigated by improved N management in agriculture. Improvements in air quality, in terms of reduced N deposition, would likely provide greater benefits for human health and inland waters than for DIN transport to the GOM. Current plans for improving WWTP capabilities may not keep pace with population growth and this situation has the potential to increase future DIN export. Taken together, the scenarios suggest that meeting the interim N reduction targets requires aggressive changes in N management, such as improving crop NRE by 43%, upgrading WWTP N-removal capabilities, and broadly modifying tile-drain systems.

## Electronic supplementary material

Below is the link to the electronic supplementary material.

Supplementary material 1 (DOCX 19562 kb)
