# Supplementary material for: Alternative futures of dissolved inorganic nitrogen export from the Mississippi River Basin: influence of crop management, atmospheric deposition, and population growth
Source: Biogeochemistry. 2017 Apr 10;133(3):263–77. doi: 10.1007/s10533-017-0331-z (PMC6260936; doi:10.1007/s10533-017-0331-z)
Supplement: Supplementary file 1 — Supplementary material 1 (DOCX 19562 kb) [file 10533_2017_331_MOESM1_ESM.docx]

Supplemental material for

Monthly Dissolved Inorganic Nitrogen Export from the Mississippi River Basin:

A New, Loosely Coupled Multimedia Model

Michelle L. McCrackin^1a^, Ellen J. Cooter^2^, Robin L. Dennis^2^, John A. Harrison^1^, and
Jana E. Compton^3^

^1^School of the Environment

Washington State University

Vancouver, WA, USA

^2^Office of Research and Development, National Exposure Research Lab

U. S. Environmental Protection Agency

Research Triangle Park, North Carolina, USA

^3^Office of Research and Development, National Health and Environmental Effects Research Laboratory, Western Ecology Division

U. S. Environmental Protection Agency

Corvallis, OR, USA

^a^Current address: Baltic Sea Centre, Stockholm University, Stockholm, Sweden

^1^Corresponding author: [michelle.mccrackin@su.se](mailto:michelle.mccrackin@su.se);

There are 11 parts to this Supplemental Material:

1: Description of NEWS2_MRB_-DIN model

2: Description of EPIC model

3: Description of CMAQ model

4: Table S1. Calibrated parameters for NEWS2_MRB_-DIN

5. Table S2. Fraction of dissolved inorganic nitrogen removed from the river by denitrification (F_deN_) by basin and month

6. Table S3. Summary of NEWS2_MRB_-DIN output for all sub-basins for 2002_HIST_, 2022_BASE_, and 2022_CROP_: SummaryofNEWS2MRBOutput.csv.

7: Figure S1. Comparison of NEWS2_MRB_-DIN basin nitrogen inputs between 2002_HIST_ and year 2022 scenarios

8: Figures S2-S5. Measured and model-derived dissolved inorganic nitrogen export for sub-basins of the Mississippi River Basin

9: Figure S6. Monthly contribution of sub-basins to dissolved inorganic N export

10: Figure S7. Map of tile drainage area cover (percent) in the Mississippi River Basin.

11: Figure S8. Map of agricultural land cover (percent) in the Mississippi River Basin.

12: References for supporting information

Total number of pages: 22

Number of tables: 3

Number of figures: 8

1. Description of NEWS2-MRB model

The model structure of NEWS2-MRB is:

DIN = (((TN_diff_ - TN_tile_) × FE_ws_) + ((TN_sew_ × FE_pnt_) + TN_tile_)) × FE_riv_ (1)

where DIN is the modeled monthly export per basin (kg N km^-2^ mo^-1^).

TN_diff_ represents diffuse sources of TN that are available for transport to the river network:

TN_diff_ = TN_dep_ + (TN_BNFcrop_ + TN_fert_) × NRE + TN_BNFback_ + TN_sep_  (2)

where TN_dep_ is rate of atmospheric deposition, TN_BNFcrop_ is rate of biological N fixation by crops, TN_fert_ is rate of organic and inorganic fertilizer application, TN_BNFback_ is rate of biological N fixation in non-agricultural areas, and TN_sep_ is the rate of leakage from septic systems (all in kg N km^-2^ mo^-1^). NRE (N recovery efficiency) is the fraction (0 to 1) of applied or fixed N removed from fields by crop harvest.

TN_tile_ is excess inorganic and organic N fertilizer not taken up by crops that is available for transport on tile-drained fields:

TN_tile_ = TN_Fert_ × (1 – NRE) × Tile_area_ (3)

where Tile_area_ is the fraction of crop land where tile drains are present.

TN_sew_ is the portion of N in human excrement that enters sewage treatment systems:

TN_sew_ = TN_excr_ × F_sewer_  (4)

where TN_excr_ is N in human excrement based on a per capita excretion rate and population size and F_sewer_ is the fraction of the population connected to sewage systems

The portion of TN in sewage effluent emitted to rivers as DIN:

FE_sew_ = (1 – F_WWTP_) (5)

where F_WWTP_ is the portion of N in human waste removed by wastewater treatment systems (Van Drecht et al., 2009).

TN_sep_ is the leakage from septic systems:

TN_sep_ = TN_excr_ × (1 - F_sewer_) × F_sep_ (6)

where F_sep_ is the portion of human excrement retained in septic systems.

The fraction of diffuse N sources that is transported from soils to the river network (FE_ws_) was modeled as a function of runoff and temperature as:

FE_ws_ = FE_RO_ × (1 - F_temp_) (7)

where FE_RO_ is the fraction (0-1) of TN exported as DIN by the terrestrial portion of the catchment controlled by runoff and F_temp_ is the fraction of TN retained in the catchment as a function of temperature (McCrackin et al., 2014). FE_RO_ was calculated as:

FE_RO_ = b × (R^a^) (8)

where R is monthly runoff (m mo^-1^) and a and b are calibrated parameters that define the shape of the relationship between R and FE_RO_. F_temp_ was estimated as:

F_temp_ = d × (T/100)^c^ (9)

where T is average seasonal air temperature (°C + 8), to eliminate negative values) and c and d are calibrated parameters that define the shape of the relationship between T and F_temp_. Air temperature is divided by 100 so that values are similar in magnitude to runoff. The exponential relationship between temperature and F_temp_ reflects the exponential shape of temperature-dependent processes such as respiration (Green et al., 2004).

As in NEWS2-DIN, the fraction of diffuse and point N sources transported by rivers to the coastal zone was estimated as:

FE_riv_ = (1-F_deN_) × (1-F_Qrem_) × (1-F_res_) (10)

where riverine sinks are denitrification in the river channel (F_deN_), consumptive water use (F_Qrem_), and denitrification and sedimentation in reservoirs (F_res_). We estimated DIN removed by denitrification (F_deN_) based on as a function of basin area, whereby larger basins remove a greater portion of N due to longer travel time in the river network (Dumont et al., 2005). To account for temperature-driven variations in denitrification, we applied a Q_10_ adjustment to F_deN_ based on the difference in average air temperature relative to the maximum average monthly temperature:

F_deN_ = (m × ln(A) – n) × Q_10_ ^(Tij – Timax)/10^  (11)

where A is basin area (km^2^), and m and n (unitless) are fitted parameters (0.0605 and 0.0443, respectively) as previously determined in NEWS2-DIN. Q_10_ is 2.71 for average air temperatures <12°C and 2.54 for temperatures >12°C (Ambus, 1993) where T_ij_ is the average air temperature (°C) for the ith river basin in the jth month, and T_imax_ is the average air temperature for the month with the greatest average temperature. Where average air temperatures were < 0°C, we assumed (recognizing this to be an oversimplification) that minimum water temperatures were 4°C to reflect the likely temperature of dense water near the sediments, where denitrification is assumed to occur in rivers (Mulholland et al., 2008). Also, the maximum F_deN_ determined by Seitzinger (2002) was 0.65; to avoid extrapolation error F_deN_ values were constrained between 0 and 0.65. See Table S2 for monthly values of F_deN_ for each basin.

Nitrogen in water removed from rivers for irrigation is treated as permanently removed from the river system. The fraction of river discharge removed for consumption (FQ_rem_) was estimated as follows:

FQ_rem_ = 1–Q_act_/Q_nat_ (12)

where Q_nat_ is estimated “natural” monthly river discharge and Q_act_ is monthly river discharge after the implementation of large-scale water removal for human use for the basin as a whole (both in km^3^ mo^-1^). Both monthly Q_nat_ and Q_act_ were obtained from (Fekete et al., 2010).

Retention of DIN in reservoirs (F_res_) is modeled as:

F_res_ = 0.8845 × (h/**τ**)^-0.3677^ (13)

where h is mean reservoir depth (meters) and **τ** is the water residence time (years) for the each reservoir in the National Inventory of Dams (USACE, 2007). The aggregated F_res_ is estimated as the average retention of all reservoirs in each basin. Retention in individual basins is capped at a maximum of 0.965 as in Dumont et al. (2005).

NEWS2 model code is open source and user documentation is available at: http://www.marine.rutgers.edu/globalnews/GNE/.

Model Notation

| A | Basin area, km^2^ |
| --- | --- |
| a | Fitted coefficient defining the relationship between runoff and FE_ws_ (0 to 1) |
| b | Fitted coefficient defining the relationship between runoff and FE_ws_ (0 to 1) |
| c | Fitted coefficient defining the relationship between temperature and FE_ws_ (-1 to 0) |
| d | Fitted coefficient defining the relationship between temperature and FE_ws_ (0 to 1) |
| DIN | Dissolved inorganic export (DIN), kg N km^-2^ mo^-1^ |
| F_deN_ | Fraction of DIN lost in the basin river network due to denitrification (0 to 1) |
| F_res_ | Fraction of DIN retained in dammed reservoirs (0 to 1) |
| F_sep_ | Fraction of N retained in septic systems; in NEWS2-MRB was set to equal to 0.46 |
| F_Qrem_ | Fraction of DIN retained, owing to the anthropogenic removal of (DIN- containing) river water (0 to 1) |
| F_sewer_ | Fraction of the population connected to centralized sewage systems (0 to 1) |
| F_WWTP_ | Fraction of N removed from effluent by sewage treatment. 0.10, 0.35, and 0.80 for primary, secondary, and tertiary WWTP, respectively |
| FE_sew_ | Fraction of TN_sew_ exported by rivers as DIN (0 to 1) |
| FE_riv_ | Fraction of total point and nonpoint DIN inputs to the river that is exported as DIN (0 to 1) |
| FE_RO_ | Fraction of N from diffuse sources in the watershed that leaches to the river as DIN as a function of runoff (0 to 1) |
| FE_temp_ | Fraction of N from diffuse sources in the watershed that leaches to the river as DIN as a function of temperature (0 to 1) |
| FE_ws_ | Fraction of N from diffuse sources in the watershed that leaches to the river as DIN. Calculated as FE_temp_ x FE_RO_ (0 to 1) |
| h | reservoir depth, m |
| m | Relationship between basin area (A) and river network retention (D_deN_); in NEWS2-MRB was set to equal 0.0605 |
| n | Relationship between basin area (A) and river network retention (D_deN_); in NEWS2-MRB was set to equal 0.0443 |
| NRE | Fraction of N inputs removed by crop uptake and harvest (0 to 1) |
| Q_act_ | Q basin discharge, km^3^ mo^-1^ |
| Q_nat_ | Amount of discharge prior to the construction of dams, km^3^ mo^-1^ |
| R | Runoff, mm mo^-1^ |
| **τ** | Reservoir water residence time, month. |
| T | Temperature, ˚C |
| Tile_area_ | Fractional area with tile drainage (0 to 1) |
| TN_BNFcrop_ | Rate of biological N fixation by crops, kg N km^-2^ mo^-1^ |
| TN_BNFnat_ | Rate of biological N fixation in natural (non-agricultural areas), kg N km^-2^ mo^-1^ |
| TN_Dep_ | Rate of atmospheric N deposition, kg N km^-2^ mo^-1^ |
| TN_diff_ | N from diffuse sources that is mobilized from the watershed soils and sediments, kg N km^-2^ mo^-1^ |
| TN_fert_ | Rate of application of organic and inorganic fertilizer, kg N km_-2_ mo^-1^ |
| TN_sep_ | TN leakage from septic systems, kg N km^-2^ mo^-1^ |
| TN_sew_ | TN to waste water treatment plants, kg N km^-2^ mo^-1^ |
| TN_tile_ | TN export from fields through artificial drainage, kg N km^-2^ mo^-1^ |

2. Description of EPIC model and output

We obtained inorganic and organic fertilizer applications and crop BNF from the USDA Environmental Policy Integrated Climate (EPIC) model, a semi-empirical process-based biogeochemical model in which N added to or lost from agricultural fields responds explicitly to farm management and land use changes (Cooter et al., 2012). The version of EPIC used here is available for download as part of the Fertilizer Emission Scenario Tool for CMAQ (FEST-C) at <http://www.cmascenter.org>.

EPIC maintains soil ammonia and nitrate budgets. A crop species-specific potential growth is simulated for each day. This potential is then reduced in response to nutrient, water, temperature, aeration, and aluminum stresses. When nutrient (N) stress is indicated, EPIC is directed to make a fertilizer application. Applications are made at planting, approximately 30 days after planting, and subsequently as needed (minimum 7-day separation) until plants reach 50% of maturity.

Planting dates are dynamically determined based on the crop, the variety simulated (based on days to maturity, e.g., 90-day variety corn, 120-day variety, etc.) and year-specific temperature and soil moisture.  Planting dates are shifted randomly, based on an operations window whose size varies with “typical” farm size or management difficulty.  In general, the window is longest in the south and shortest in the north.  The modeled state-level planting date distribution are evaluated against USDA crop progress reports to validates both the area being planted as well as the rate at which planted area changes over time within the state. Evaluation of these EPIC functions is provided in Cooter et al. (2012) and Williams et al. (2012).

It is assumed that projected economically driven demand will induce extensification in crop distribution on existing agricultural lands (including Conservation Reserve Program), but the amount of agricultural land is fixed at National Land Classification Dataset (Homer et al., 2007) version 2011 levels (http://www.mrlc.gov). Crop yield response to agricultural technology advances and intensification projected by the coupled market sector model are modeled in EPIC by increasing the harvest index parameters in EPIC (i.e., the ratio of the economic yield and above ground biomass).

EPIC also estimates BNF for legume crop species such as alfalfa, soybeans, peanuts, and beans as a function of a calculated rate considering growth stage, soil water content, soil N content, crop N uptake rate, and nitrate content in the soil to the root depth (Williams et al., 2012). Crop response to CO_2_ is modeled primarily through its influence on the rate of energy to biomass conversion (Monteith and Moss, 1977, Stockle et al., 1992). Simulated daily weather was from the Weather Research Forecast (WRF) model and held constant between years 2002 and 2022 (Skamarock et al., 2009).

3. Description of CMAQ model and output

We obtained monthly rates of atmospheric N deposition from fossil-fuel combustion and agricultural sources from the CMAQ version 5.0.2 with Bi-Directional Ammonia (Bash et al., 2013, Cooter et al., 2012) run at a 12 km rectangular grid resolution over the continental U.S., southern Canada and Northern Mexico (i.e., US1 domain). There are three CMAQ simulations that parallel those scenarios described for EPIC. The CMAQ scenarios are all driven by an hourly time-step version of the same 2002 WRF (35-layer configuration, version 3.4) simulated weather conditions in order to isolate nitrogen export changes driven by agricultural technology, intensification and extensification, from those driven by uncertain future weather.

Each CMAQ scenario requires an appropriate anthropogenic emissions inventory. The 2002 CMAQ simulation used the National Emissions Inventory (2002 NEI) version 2 (<https://www3.epa.gov/ttn/chief/net/2002inventory.html>). The 2022_BASE_ simulation used the Energy Information Administration’s (EIA) Annual Energy Outlook (AEO) 2007 inventory (EIA, 2007) which represents emissions associated with ethanol volumes that were projected in 2022 in the absence of the Energy Independence and Security Act (EISA) renewable Fuel mandates (USEPA, 2010). The emission projections used in both 2022_BASE_ and 2022_CROP_ scenarios reflect the full implementation of the Clean Air Interstate Rule and Tier 2 light-duty vehicle standards, on road heavy duty engine standards, and existing non-road engine standards.

The 2022_CROP_ scenario begins with the AEO inventory, to which we apply hypothetical growth and control factors to account for changes in pollutants by technology, sector, and region reflecting ethanol and biodiesel plant additions, ethanol transfer additions, changes in mobile source emissions, and other changes in point and non-point emissions. It assumes a demand for 10,000 million gallons of cellulosic-based fuel, and demand for 18,000 million gallons of starch-based fuel. The cellulosic and starch-based demands and production are hypothetical levels used to drive the FAPRI/CARD integrated energy and agricultural model. All additional biomass-crop demands modeled in Elobeid et al. (2013) are assumed to be met through increased production and processing of corn and corn-stover feedstocks. 2022_CROP_ is intended to model a more corn-starch and corn stover intensive fuel production scenario in order to examine the environmental impacts of this alternative scenario on major corn-producing regions. In order to translate the fuel production scenarios from Elobeid et al. (2013) into emission changes, modifications were made to the non-point input files for biodiesel, cellulosic, corn ethanol plants, as well as to capture changes in levels of VOCs from ethanol transfers. Corn-based production and associated emissions were assumed to grow in the same county, in accordance with the increases in fuel production levels. For the cellulosic production facilities, however, the size, number, as well as location, of these facilities were changed based on production estimates from the integrated energy and agricultural markets model. This led to greater concentration of cellulosic facilities in counties with high production levels and higher corn-stover removals limits. For the most part, the facilities are located near feedstock sources in the Upper Mississippi and Ohio river basins. Production facility emissions associated with increased facility capacity or the siting of new facilities are provided to CMAQ *via* the modified emissions input files.

CMAQ simulates the response of total atmospheric N-deposition associated with these emission sources for each 12 x 12 km model grid-cell. Monthly grid cell average depositions were then computed and adjusted for precipitation bias as described in Appel et al. (2011) using Parameter-elevation Relationships on Independent Slopes Model (PRISM) data (<http://www.prism.oregonstate.edu>) for each river basin. Simulated daily weather was from the Weather Research Forecast (WRF) model and held constant between years 2002 and 2022 (Skamarock et al., 2009).

4. Table S1: Calibrated parameters for NEWS2_MRB_-DIN model. Values are from 1,000 resampling iterations. Standard deviation is SD.

| Parameter | Mean | SD |
| --- | --- | --- |
| a | 0.78 | 0.04 |
| b | 0.97 | 0.07 |
| c | 0.78 | 0.37 |
| d | 0.03 | 0.04 |

|  | Month | | | | | | | | | | | |
| --- | --- | --- | --- | --- | --- | --- | --- | --- | --- | --- | --- | --- |
| Basin | 1 | 2 | 3 | 4 | 5 | 6 | 7 | 8 | 9 | 10 | 11 | 12 |
| Arkansas-Red  River Basin | 0.07 | 0.07 | 0.07 | 0.21 | 0.32 | 0.50 | 0.65 | 0.60 | 0.40 | 0.23 | 0.11 | 0.07 |
| Missouri River Basin | 0.11 | 0.11 | 0.11 | 0.15 | 0.29 | 0.47 | 0.65 | 0.59 | 0.35 | 0.18 | 0.11 | 0.11 |
| Ohio River Basin | 0.15 | 0.15 | 0.15 | 0.25 | 0.39 | 0.55 | 0.65 | 0.61 | 0.46 | 0.27 | 0.17 | 0.15 |
| Upper Mississippi River Basin | 0.10 | 0.10 | 0.10 | 0.16 | 0.32 | 0.52 | 0.65 | 0.57 | 0.37 | 0.19 | 0.10 | 0.10 |
| Mississippi River Basin | 0.10 | 0.10 | 0.10 | 0.17 | 0.32 | 0.50 | 0.65 | 0.59 | 0.38 | 0.20 | 0.10 | 0.10 |

5. Table S2. Fraction of dissolved inorganic nitrogen removed from the river by denitrification (F_deN_) by basin and month.

7. Figure S1: Land-based nitrogen inputs used in NEWS2_MRB_-DIN model runs for 2002_HIST_ (black bars) and two year 2022 scenarios (gray bars) for the Mississippi River Basin and sub-basins.





8. Figure S2. Comparison of measured (gray bars) and model-derived (lines) dissolved inorganic nitrogen (DIN) export (kg N km^-2^ mo^-1^) for the Arkansas-Red River Basin. Box plot shows median value (horizontal line), 25^th^ and 75^th^ percentile (box outline), 10^th^ and 90^th^ percentile (error bars), and 5^th^ and 95^th^ percentile (solid point).





Figure S3. Comparison of measured (gray bars) and model-derived (lines) dissolved inorganic nitrogen (DIN) export (kg N km^-2^ mo^-1^) for the Missouri River Basin. Box plot shows median value (horizontal line), 25^th^ and 75^th^ percentile (box outline), 10^th^ and 90^th^ percentile (error bars), and 5^th^ and 95^th^ percentile (solid point).





Figure S4. Comparison of measured (gray bars) and model-derived (lines) dissolved inorganic nitrogen (DIN) export (kg N km^-2^ mo^-1^) for the Ohio-Tennessee River Basin. Box plot shows median value (horizontal line), 25^th^ and 75^th^ percentile (box outline), 10^th^ and 90^th^ percentile (error bars), and 5^th^ and 95^th^ percentile (solid point).





Figure S5. Comparison of measured (gray bars) and model-derived (lines) dissolved inorganic nitrogen (DIN) export (kg N km^-2^ mo^-1^) for the Upper Mississippi River Basin. Box plot shows median value (horizontal line), 25^th^ and 75^th^ percentile (box outline), 10^th^ and 90^th^ percentile (error bars), and 5^th^ and 95^th^ percentile (solid point).





9. Figure S6. Model-derived contribution of sub-basins to dissolved inorganic N export (DIN, kg mo^-1^) in the Mississippi River Basin for year 2002 (top panel), year 2022_BASE_ (middle panel), and year 2022_CROP_ (bottom panel). Values for the Lower Mississippi River Basin (LMRB) were estimated by difference; negative values indicate DIN sink.

2002_HIST_

2022_BASE_

2022_CROP_


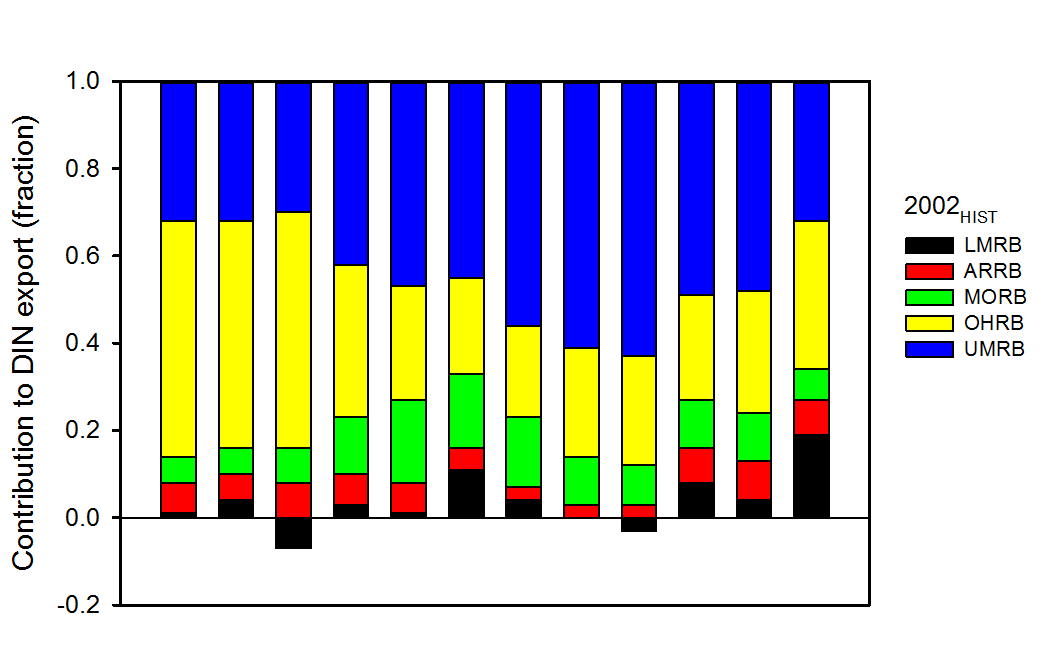

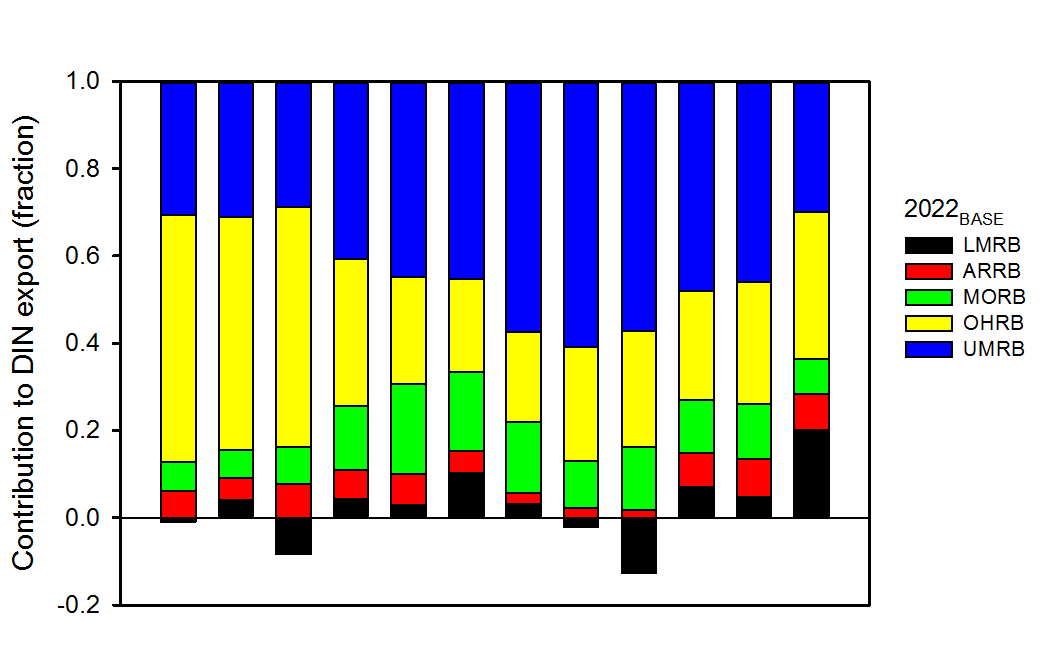

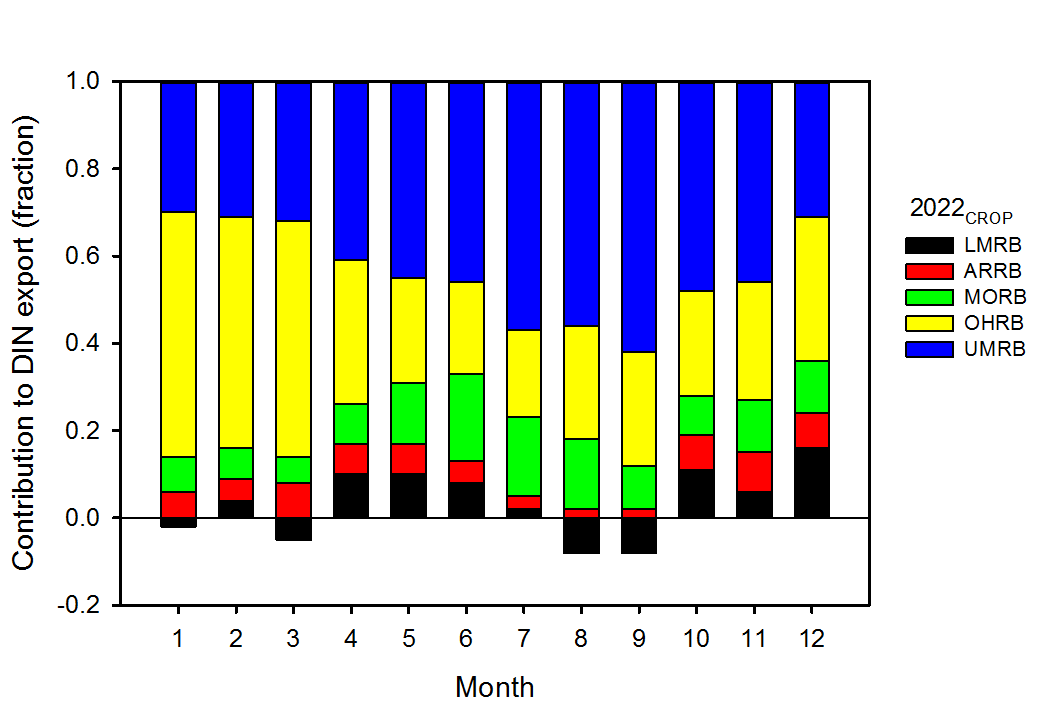





10. Figure S7. Map of agricultural land cover (percent) in the Mississippi River Basin.


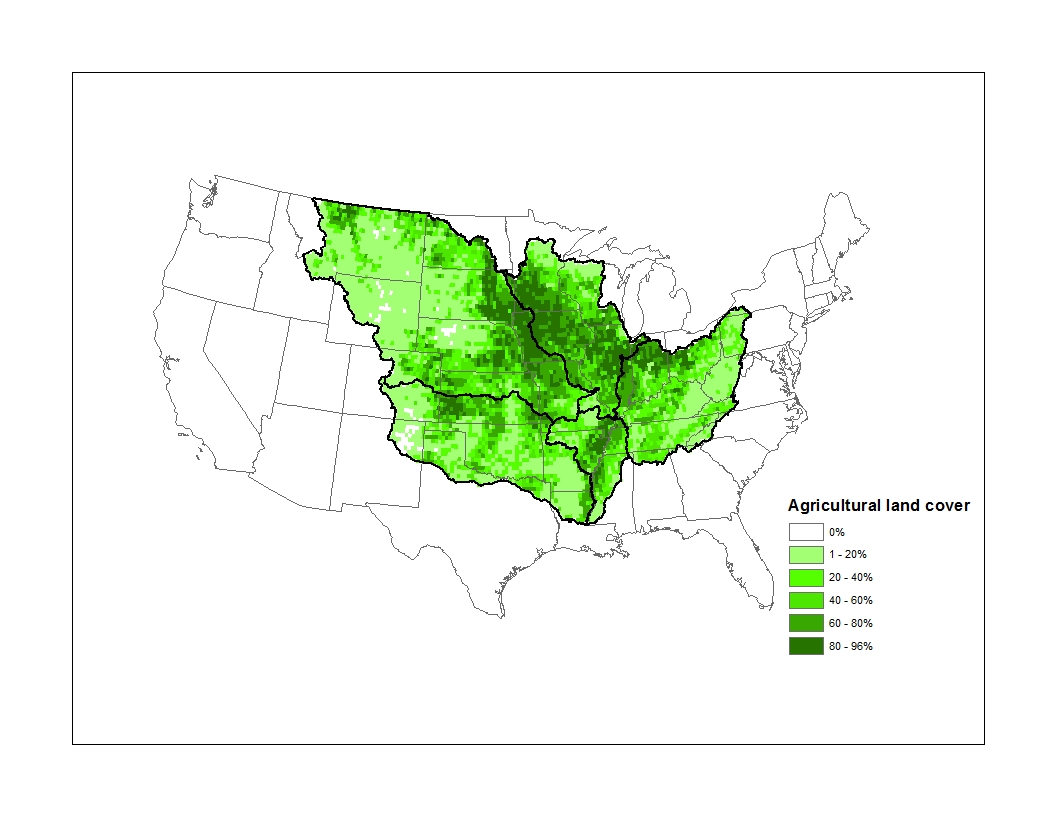


11. Figure S8. Map of tile drainage area cover (percent) in the Mississippi River Basin. Modified from Sugg (2007).


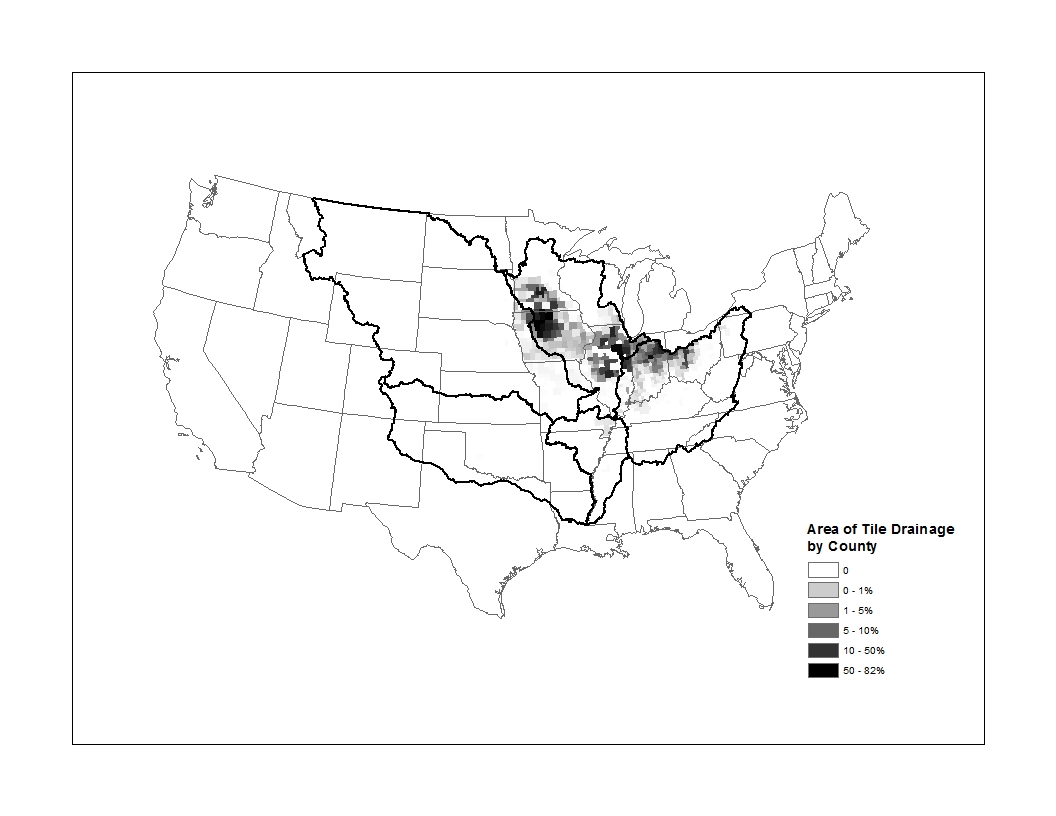


12. References for supporting information

Ambus, P. 1993. Control of denitrification enzyme-activity in a streamside soil. FEMS Microbiology Ecology 102: 225-234.

Appel, K.W., K.M. Foley, J. Bash, R.W. Pinder, R.L. Dennis, D.J. Allen, et al. 2011. A multi-resolution assessment of the Community Multiscale Air Quality (CMAQ) model v4.7 wet deposition estimates for 2002-2006. Geoscientific Model Development 4: 357-371. doi:10.5194/gmd-4-357-2011.

Bash, J.O., E.J. Cooter, R.L. Dennis, J.T. Walker and J.E. Pleim. 2013. Evaluation of a regional air-quality model with bidirectional NH3 exchange coupled to an agroecosystem model. Biogeosciences 10: 1635-1645. doi:10.5194/bg-10-1635-2013.

Cooter, E., J. Bash, V. Benson and L. Ran. 2012. Linking Agricultural Crop Management and Air Quality Models for Regional to National-Scale Nitrogen Assessments. Biogeosciences 9: 6095-6127.

Dumont, E., J. Harrison, C. Kroeze, E.J. Bakker and S. Seitzinger. 2005. Global distribution and sources of dissolved inorganic nitrogen export to the coastal zone: Results from a spatially explicit, global model. Global Biogeochemical Cycles 19: GB4S02 doi:10.1029/2005GB002488.

EIA. 2007. Energy Information Administration Annual Energy Outlook 2007 with Projections to 2030 Report Number: DOE/EIA-0380(2007). <http://www.eia.gov/oiaf/archive/aeo0307/pdf/0383(2007).pdf>.

Elobeid, A., S. Tokgoz, R. Dodder, T. Johnson, O. Kaplan, L. Kurkalova, et al. 2013. Integration of agricultural and energy system models for biofuel assessment. Environmental Modelling & Software 48: 1-16. doi:10.1016/j.envsoft.2013.05.007.

Fekete, B.M., D. Wisser, C. Kroeze, E. Mayorga, L. Bouwman, W.M. Wollheim, et al. 2010. Millennium Ecosystem Assessment scenario drivers (1970-2050): Climate and hydrological alterations. Global Biogeochemical Cycles 24: GB0A12, doi:10.1029/2009GB003593.

Green, P.A., C.J. Vörösmarty, M. Meybeck, J.N. Galloway, B.J. Peterson and E.W. Boyer. 2004. Pre-industrial and contemporary fluxes of nitrogen through rivers: a global assessment based on typology. Biogeochemistry 68: 71-105.

Homer, C., J. Dewitz, J. Fry, M. Coan, N. Hossain, C. Larson, et al. 2007. Completion of the 2001 National Land Cover Database for the Conterminous United States. Photogrammetric Engineering and Remote Sensing 73: 337-341.

McCrackin, M.L., J.A. Harrison and J.E. Compton. 2014. Future Riverine Nitrogen Export to Coastal Regions in the United States: Prospects for Improving Water Quality. Journal of Environment Quality 0: 0. doi:10.2134/jeq2014.02.0081.

Monteith, J.L. and C.J. Moss. 1977. Climate and the Efficiency of Crop Production in Britain [and Discussion]. Philos Trans R Soc Lond B Biol Sci 281: 277-294.

Mulholland, P.J., A.M. Helton, G.C. Poole, R.O. Hall, S.K. Hamilton, B.J. Peterson, et al. 2008. Stream denitrification across biomes and its response to anthropogenic nitrate loading. Nature 452: 202-206.

Seitzinger, S.P., C. Kroeze, A.F. Bouwman, N. Caraco, F. Dentener and R.V. Styles. 2002. Global patterns of dissolved inorganic and particulate nitrogen inputs to coastal systems: Recent conditions and future projections. Estuaries 25: 640-655. doi:10.1007/bf02804897.

Skamarock, W.C., J.B. Klemp, J. Dudhia, D.O. Gill, D.M. Barker, M.G. Duda, et al. 2009. A description of the Advanced Research WRF version 3, National Center for Atmospheric Research Boulder, 125.

Stockle, C.O., J.R. Williams, N.J. Rosenberg and C.A. Jones. 1992. A method for estimating the direct and climatic effects of rising atmospheric carbon-dioxide on growth and yield of crops .1. Modification of the epic model for climate change analysis. Agricultural Systems 38: 225-238. doi:10.1016/0308-521x(92)90067-x.

Sugg, Z. 2007. Assessing U.S. Farm Drainage: Can GIS Lead to Better Estimates of Subsurface Drainage Extent? World Resources Institiute, Washington DC, USA. <http://www.wri.org/publication/assessing-us-farm-drainage>.

USACE. 2007. US Army Core of Engineers. 2007 National Inventory of Dams. [http://nid.usace.army.mil/](http://nid.usace.army.mil).

USEPA. 2010. RFS2 Emissions Inventory for Air Quality Modeling Technical Support Document, Report Number EPA-420-R-10-005. . <https://www.epa.gov/sites/production/files/2015-2008/documents/2420r10005.pdf>.

Van Drecht, G., A.F. Bouwman, J. Harrison and J.M. Knoop. 2009. Global nitrogen and phosphate in urban wastewater for the period 1970 to 2050. Global Biogeochemical Cycles 23: GB0A03 doi:10.1029/2009GB003458.

Williams, J.R., R.C. Izaurralde and E.M. Steglich. 2012. Agricultural Policy/Environmental Extender Model Theoretical Documentation version 0806. Downloadable from: [http://epicapex.tamu.edu/](http://epicapex.tamu.edu).
